# On the Densest Packing of Polycylinders in Any Dimension

**DOI:** 10.1007/s00454-016-9766-6

**Published:** 2016-03-01

**Authors:** Wöden Kusner

**Affiliations:** Institute of Analysis and Number Theory, Graz University of Technology, Steyrergasse 30/II, 8010 Graz, Austria

**Keywords:** Polycylinders, Packing, Density, Slicing

## Abstract

Using transversality and a dimension reduction argument, a result of Bezdek and Kuperberg is applied to polycylinders, showing that the optimal packing density of $\mathbb {D}^2\times \mathbb {R}^n$ equals $\pi /\sqrt{12}$ for all natural numbers *n*.

## Introduction

Open and closed Euclidean unit *n*-balls will be denoted by $\mathbb {B}^n$ and $\mathbb {D}^n$ respectively. The closed unit interval is denoted by $\mathbb {I}$. A general polycylinder *C* is a set congruent to $\prod _{i=1}^{i=m}\lambda _i\mathbb {D}^{k_i}$ in $\mathbb {R}^{ k_1+\dots + k_m}$, where $\lambda _i$ is in $[0,\infty ]$. For this article, the term polycylinder refers to the special case of an infinite polycylinder over a two-dimensional disk of unit radius. A *polycylinder* is a set congruent to $\mathbb {D}^2 \times \mathbb {R}^n$ in $\mathbb {R}^{n+2}$. A *polycylinder packing of*$\mathbb {R}^{n+2}$ is a family $\mathscr {C} = \{C_i\}_{i \in I}$ of polycylinders $C_i \subset \mathbb {R}^{n+2}$ with mutually disjoint interiors. The *upper density*$\delta ^+ (\mathscr {C})$ of a packing $\mathscr {C}$ of $\mathbb {R}^n$ is defined to be

$$\begin{aligned} \delta ^+ (\mathscr {C}) = \limsup _{r\rightarrow \infty }\tfrac{ {{\mathrm{Vol}}}(\mathscr {C}\cap r\mathbb {B}^n)}{{{\mathrm{Vol}}}(r\mathbb {B}^n)}. \end{aligned}$$

The *upper packing density*$\delta ^+ (C)$ of an object *C* is the supremum of $\delta ^+ (\mathscr {C})$ over all packings $\mathscr {C}$ of $\mathbb {R}^n$ by *C*.

This article proves the following sharp bound for the packing density of infinite polycylinders:

### **Theorem 1**

$\delta ^+(\mathbb {D}^2 \times \mathbb {R}^n) = \pi /\sqrt{12}$ for all natural numbers *n*.

Theorem 1 generalizes a result of Bezdek and Kuperberg [1] and improves on results that may be computed using a method of Fejes Tóth and Kuperberg [3], cf. [2, 5]; it gives some of the first sharp upper bounds for packing density in high dimensions.

## Transversality

This section introduces the required transversality arguments from affine geometry. A *d-flat* is a *d*-dimensional affine subspace of $\mathbb {R}^n$. The *parallel dimension*$\mathrm{dim}_\parallel \{F,\dots , G\}$ of a collection of flats $\{F, \dots , G\}$ is the dimension of their maximal parallel sub-flats. The notion of parallel dimension can be interpreted in several ways, allowing a modest abuse of notation.

- For a collection of flats $\{F, \dots , G\}$, consider their tangent cones at infinity $\{F_\infty , \dots , G_\infty \}$. The parallel dimension of $\{F, \dots , G\}$ is the dimension of the intersection of these tangent cones. This may be viewed as the limit of a rescaling process $\mathbb {R}^n \rightarrow r\mathbb {R}^n$ as *r* tends to 0, leaving only the scale-invariant information.
- For a collection of flats $\{F, \dots , G\}$, consider each flat as a system of linear equations. The corresponding homogeneous equations determine a collection of linear subspaces $\{F_\infty , \dots , G_\infty \}$. The parallel dimension is the dimension of their intersection $F_\infty \,\cap \dots \cap \, G_\infty$.

Two disjoint *d*-flats are *parallel* if their parallel dimension is *d*, that is, if every line in one is parallel to a line in the other.

### **Lemma 1**

A pair of disjoint *n*-flats in $\mathbb {R}^{n+k}$ with $n\ge k$, has parallel dimension strictly greater than $n-k.$

### *Proof*

Let *F* and *G* be such a pair. By homogeneity of $\mathbb {R}^{n+k}$, let $F=F_\infty .$ As $F_\infty$ and *G* are disjoint, *G* contains a non-trivial vector $\mathbf{v}$ such that $G = G_\infty +\mathbf{v}$ and $\mathbf{v}$ is not in $F_\infty + G_\infty .$ It follows that

$$\begin{aligned} \mathrm{dim} (\mathbb {R}^{n+k})\ge & {} \mathrm{dim}\big (F_\infty + G_\infty + {{\mathrm{span}}}(\mathbf{v})\big )> \mathrm{dim}(F_\infty + G_\infty )\\= & {} \mathrm{dim} (F_\infty ) + \mathrm{dim} (G_\infty ) - \mathrm{dim} (F_\infty \cap G_\infty ). \end{aligned}$$

Count dimensions to find $$n+k > n +n - \mathrm{dim}_\parallel (F_\infty ,G_\infty ).$$$$\square $$

### **Corollary 1**

A pair of disjoint *n*-flats in $\mathbb {R}^{n+2}$ has parallel dimension at least $n-1$.

## Dimension Reduction

### Pairwise Foliations

The *core*$a_i$ of a polycylinder $C_i$ congruent to $\mathbb {D}^2 \times \mathbb {R}^n$ in $\mathbb {R}^{n+2}$ is the distinguished *n*-flat defining $C_i$ as the set of points at most distance 1 from $a_i$. In a packing $\mathscr {C}$ of $\mathbb {R}^{n+2}$ by polycylinders, Corollary 1 shows that, for every pair of polycylinders $C_i$ and $C_j$, one can choose parallel $(n-1)$-dimensional subflats $b_i \subset a_i$ and $b_j \subset a_j$ and define a product foliation

$$\begin{aligned} \mathscr {F}^{b_i,b_j}:\mathbb {R}^{n+2} \rightarrow \mathbb {R}^{n-1} \times \mathbb {R}^3 \end{aligned}$$

with $\mathbb {R}^3$ leaves that are orthogonal to $b_i$ and to $b_j$. Given a point *x* in $a_i$, there is a distinguished $\mathbb {R}^3$ leaf $F_x^{b_i,b_j}$ that contains the point *x*. The foliation $\mathscr {F}^{b_i,b_j}$ restricts to foliations of $C_i$ and $C_j$ with right-circular-cylinder leaves.

### The Dirichlet Slice

In a packing $\mathscr {C}$ of $\mathbb {R}^{n+2}$ by polycylinders, the *Dirichlet cell*$D_i$ associated with a polycylinder $C_i$ is the set of points in $\mathbb {R}^{n+2}$ which lie no further from $C_i$ than from any other polycylinder in $\mathscr {C}$. The Dirichlet cells of a packing partition $\mathbb {R}^{n+2},$ as $C_i \subset D_i$ for all polycylinders $C_i$. To bound the density $\delta ^+(\mathscr {C})$, it is enough to fix an *i* in *I* and consider the density of $C_i$ in $D_i$.

Consider the following slicing of the Dirichlet cell $D_i$. Given a fixed polycylinder $C_i$ in a packing $\mathscr {C}$ of $\mathbb {R}^{n+2}$ by polycylinders and a point *x* on the core $a_i$, the plane $p_x$ is the 2-flat orthogonal to $a_i$ and containing the point *x*. The *Dirichlet slice*$d_x$ is the intersection of $D_i$ and $p_x.$

Note that $p_x$ is a sub-flat of $F_x^{b_i,b_j}$ for all *j* in *I*.

### Bezdek–Kuperberg Bound

For any point *x* on the core $a_i$ of a polycylinder $C_i$, the results of Bezdek and Kuperberg [1] apply to the Dirichlet slice $d_x$.

#### **Lemma 2**

A Dirichlet slice is convex and, if bounded, a parabola-sided polygon.

#### *Proof*

Construct the Dirichlet slice $d_x$ as an intersection. Define $d^j$ to be the set of points in $p_x$ which lie no further from $C_i$ than from $C_j$. Then the Dirichlet slice $d_x$ is realized as

$$\begin{aligned} d_x = \big \{\bigcap _{j\in I} d^j \big \}. \end{aligned}$$

Each arc of the boundary of $d_x$ in $p_x$ is given by an arc of the boundary of some $d^j$ in $p_x.$ The boundary of $d^j$ in $p_x$ is the set of points in $p_x$ equidistant from $C_i$ and $C_j.$ Since the foliation $\mathscr {F}^{b_i,b_j}$ is a product foliation, the arc of the boundary of $d^j$ in $p_x$ is also the set of points in $p_x$ equidistant from the leaf $C_i \cap F_x^{b_i,b_j}$ of $\mathscr {F}^{b_i,b_j}|_{C_i}$ and the leaf $C_j \cap F_x^{b_i,b_j}$ of $\mathscr {F}^{b_i,b_j}|_{C_j}$. This reduces the analysis to the case of a pair of cylinders in $\mathbb {R}^3.$ From [1], it follows that $d^j$ is convex and the boundary of $d_j$ in $p_x$ is a parabola; the intersection of such sets $d^j$ in $p_x$ is convex, and a parabola-sided polygon if bounded. $\square$

Let $S_x(r)$ be the circle of radius *r* in $p_x$ centered at *x*.

#### **Lemma 3**

The vertices of $d_x$ are not closer to $S_x(1)$ than the vertices of a regular hexagon circumscribed about $S_x(1).$

#### *Proof*

A vertex of $$d_x$$ occurs where three or more polycylinders are equidistant, so the vertex is the center of a $$(n+2)$$-ball *B* tangent to three polycylinders. Thus *B* is tangent to three disjoint unit $$(n+2)$$-balls $$B_1$$, $$B_2$$, $$B_3$$. By projecting into the affine hull of the centers of $$B_1$$, $$B_2$$, $$B_3$$, it is immediate that the radius of *B* is no less than $$2/\sqrt{3} -1.$$$$\square $$

#### **Lemma 4**

Let *y* and *z* be points on the circle $S_x(2/\sqrt{3})$. If each of *y* and *z* is equidistant from $C_i$ and $C_j$, then the angle *yxz* is smaller than or equal to $2\arccos (\sqrt{3} -1) = 85.8828\dots ^\circ .$

#### *Proof*

Following [1, 4], the existence of a supporting hyperplane of $C_i$ that separates ${{\mathrm{int}}}(C_i)$ from ${{\mathrm{int}}}(C_j)$ suffices. $\square$

In [1], it is shown that planar objects satisfying Lemmas 2, 3 and 4 have area no less than $\sqrt{12}.$ As the bound holds for all Dirichlet slices, it follows that $\delta ^+(\mathbb {D}^2\times \mathbb {R}^n) \le \pi /\sqrt{12}$ in $\mathbb {R}^{n+2}.$ The product of the dense disk packing in the plane with $\mathbb {R}^n$ gives a polycylinder packing in $\mathbb {R}^{n+2}$ that achieves this density. Combining this with the result of Thue [6] for $n=0$ and the result of Bezdek and Kuperberg [1] for $n=1$, Theorem 1 follows.
